# Exploring Common Culinary Herbs and Spices as Potential Anti-Quorum Sensing Agents

**DOI:** 10.3390/nu11040739

**Published:** 2019-03-29

**Authors:** Sekelwa Cosa, Sushil Kumar Chaudhary, Weiyang Chen, Sandra Combrinck, Alvaro Viljoen

**Affiliations:** 1 Department of Pharmaceutical Sciences, Tshwane University of Technology, Private Bag X680, Pretoria 0001, South Africa; sekelwa.cosa@gmail.com (S.C.); sushpharma525@gmail.com (S.K.C.); chenw@tut.ac.za (W.C.); combrincks@tut.ac.za (S.C.); 2 SAMRC Herbal Drugs Research Unit, Tshwane University of Technology, Private Bag X680, Pretoria 0001, South Africa

**Keywords:** antimicrobial, anti-quorum sensing, *Apium graveolens*, HPTLC-bio-autography, LC-MS, 3-*n*-butyl-4,5-dihydrophthalide, sedanenolide

## Abstract

Quorum sensing controls bacterial pathogenesis and virulence; hence, interrupting this system renders pathogenic bacteria non-virulent, and presents a novel treatment for various bacterial infections. In the search for novel anti-quorum sensing (AQS) compounds, 14 common culinary herbs and spices were screened for potential antipathogenicity activity against *Chromobacterium violaceum* ATCC 12472. Extracts of *Glycyrrhiza glabra* (liquorice), *Apium graveolens* (celery), *Capsicum annuum* (cayenne pepper) and *Syzygium anisatum* (aniseed) demonstrated good AQS potential, yielding opaque halo zones ranging from 12–19 mm diameter at sub-minimum inhibitory concentrations (0.350–4.00 mg/mL). For the same species, the percentage reduction in violacein production ranged from 56.4 to 97.3%. Zones with violacein inhibitory effects were evident in a celery extract analysed using high performance thin layer chromatography-bio-autography. The major active compound was isolated from celery using preparative-high performance liquid chromatography-mass spectrometry and identified using gas chromatography-mass spectrometry (GC-MS) as 3-*n*-butyl-4,5-dihydrophthalide (sedanenolide). Potent opaque zones of inhibition observed on the HPTLC-bio-autography plate seeded with *C. violaceum* confirmed that sedanenolide was probably largely responsible for the AQS activity of celery. The bacteriocidal properties of many herbs and spices are reported. This study, however, was focussed on AQS activity, and may serve as initial scientific validation for the anti-infective properties ascribed to several culinary herbs and spices.

## 1. Introduction

Herbs and spices play a prominent role in the traditional culinary practices of many cultures and are an indispensable part of their daily diets. Culinary herbs and spices (in the form of leaves, roots, bark, berries, buds, seeds, stigmas or flowers) impart flavour, aroma and colour to dishes, and have been used for centuries for the preservation of food, including meats, sauces, vegetables, desserts, and in wine making [1,2]. Their main effects are to retard microbial growth and lipid oxidation during storage. Several herbs and spices, such as clove, cinnamon, oregano and rosemary, are reported to have significant antimicrobial [3], anti-oxidant [4,5] and/or anticarcinogenic activities. The literature reports a clear relationship between the anti-oxidant activity, antibacterial activity and total phenolic content of some herbs and spices [2,6,7]. However, non-phenolic compounds may also contribute to the antimicrobial effect of plants, for example 1′-acetoxychavicol acetate in galangal and allyl isothiocyanate in horseradish [8]. 

Although herbs and spices are minor ingredients of foods [9] and exhibit inferior activity when compared to antibiotic drugs, they have fewer side effects and are classified as “Generally Recognised As Safe” (GRAS) by the United States Food and Drug Administration (USFDA). Many of their bioactive compounds have been investigated extensively for the potential prevention and treatment of various diseases [10]. Beneficial human health-promoting properties include the treatment of toothache, fever, and pain (clove), nervous system conditions and stomach/intestinal infections (cinnamon), the use as an antiseptic and diuretic (mustard, garlic), application to accelerate wound healing (basil), and anticancer effects (turmeric) [11,12]. Some culinary herbs and spices possess anti-adhesive properties, meaning that they contain compounds that prevent the adhesion of microbes to the host tissue, thus preventing primary infection [11]. Although the biological activities of several culinary herbs and spices have been reported, their anti-quorum sensing (AQS) properties have remained unexplored. 

Quorum sensing (QS) regulates some communal behaviours that involve significant phenomena such as bioluminescence, swarming, biofilm formation, motility, sporulation, antibiotic production and the expression of virulence factors, including the production of lytic enzymes, toxins, siderophores and adhesion molecules [13,14,15] in several bacteria. Virulence and pathogenicity in many clinically relevant bacterial pathogens are controlled by QS [16,17]. An approach based on obstructing microbial communication has emerged as an effective strategy to impede cooperative actions and reduce pressure on bacteria to develop resistance [18]. Compounds that interfere with the QS system to attenuate bacterial pathogenicity are termed anti-quorum sensing (AQS) compounds. Although such compounds do not kill bacteria, or inhibit their growth, bacteria are less likely to develop resistance towards them, in contrast to existing antibiotics [19,20]. Effective AQS agents are required to be chemically stable, exert negligible toxicity, and have a low molecular weight and a high degree of specificity for the signal receptor [15]. 

Hypothetically, it is reasonable to speculate that herbs and spices may contain QS-inhibiting phytochemicals, in view of the vast array of potentially bioactive secondary metabolites produced by plants. The objective of this study was therefore to investigate the potential of selected common culinary herbs and spices as AQS agents. Fourteen herbs and spices were screened for AQS activity against *Chromobacterium violaceum*, a Gram-negative bacterium, which produces a violacein pigment in response to QS-regulated gene expression. In *C. violaceum*, the production of violacein is regulated by genes arranged in an operon that is mediated by the acylhomoserine lactone (AHL) QS system [21]. The strain is generally accepted as a convenient model to assay for AQS agents.

## 2. Materials and Methods 

### 2.1. Plant Materials and Extraction

Fourteen herbs and spices (Table 1), selected for anti-QS screening, were purchased from Warren Chemicals, South Africa. The specific plant part used as a herb or spice, for example, the root of horseradish, was obtained. Reference material has been retained in the Department of Pharmaceutical Sciences at Tshwane University of Technology. The criterion for choosing the plants was based on their traditional use associated with antimicrobial properties. Portions (5.0 g) of powdered herb material were individually extracted with solvents (50.0 mL) representing a range of polarities (water, methanol, ethyl acetate and dichloromethane), for 48 h at room temperature. The respective extracts were filtered using Whatman No. 1 filter paper. The residual material was returned for re-extraction, whereafter the combined corresponding crude extracts were air-dried in a fume hood for up to three days, depending on the extract, or freeze-dried (water extract) (SP Scientific freeze dryer USA) and the residues weighed. Stock solutions of each crude extract (100 mg/mL) were prepared using dimethyl sulfoxide (1% DMSO; Merck, Johannesburg, South Africa) and further diluted to yield final concentrations ranging from 0.350 to 7.00 mg/mL. The sterility of the extracts was verified by streaking them on Luria Bertani (LB) agar, followed by incubation at 37 °C for 24 h. The percentage yields of different extracts were calculated using the formula:

$$Percentage\;yield\;(\%)=\frac{dry\;crude\;extract}{dry\;\mathrm{initial}\;\mathrm{plant}\;\mathrm{material}\;\mathrm{before}\;\mathrm{extraction}}\times 100$$

### 2.2. Test Bacteria and Culture Media

Biosensor strain *Chromobacterium violaceum* ATCC 12472, a wild-type strain producing a QS-controlled purple pigment, violacein, was used for the qualitative and quantitative determination of QS inhibition. An active bacterial culture was prepared in LB broth and agar (1% tryptone, 0.5% yeast extract, 1% NaCl and 1.5% agar) medium at 30 °C for 24 h with shaking. Cultures were maintained in LB agar at 4 °C. 

### 2.3. Determination of the Antimicrobial Activities of Extracts

Antimicrobial susceptibility of the 56 extracts was determined using the agar well diffusion method [39]. *C. violaceum* was grown on LB agar plates overnight at 30 °C, where after; the turbidity of the cell suspensions was adjusted using sterile deionised water, to a 0.5 McFarland standard equivalent. These were used to inoculate Mueller-Hinton (MH) agar plates by swabbing over the entire surface. After boring wells into the agar with a sterile 6 mm diameter cork borer, the wells were filled with 0.350, 1.75, 3.85 and 7.00 mg/mL of each of the extracts, taking care to prevent spillage onto the surface of the agar medium. Plates were left on the laboratory bench to allow proper diffusion of the extract into the medium. After incubating the plates at 30 °C for 24 h, the zones of inhibition were measured. Eugenol (0.066 mg/mL) and ciprofloxacin (5 µg/mL) were used as positive controls, while 1% DMSO was used as the negative control. The diameter of the inhibition zone was interpreted as follows: Susceptible (S) ≥ 15 mm, Intermediate (I) = 11–14 mm, and Resistant (R) ≤ 10 mm as described by the Clinical and Laboratory Standards Institute [40].

### 2.4. Determination of the Minimum Inhibitory Concentration

The minimum inhibitory concentrations (MIC) of the crude extracts against *C. violaceum* were determined in triplicate in sterile, disposable flat-bottom 96-well microtitre plates, using the broth microdilution method as previously described [41]. Nutrient broth (100 μL) was introduced into each of the 96 wells. Two-fold serial dilution of the plant extracts (32 mg/mL) was carried out by placing a 50 μL portion of each test solution into a well in the first row, and after mixing, transferring a 50 µL portion of each well content to the adjacent well in the next row, to yield nine test concentrations ranging from 8.00 to 0.0620 mg/mL for each solvent extract. Bacterial suspension (50 μL) was subsequently added to each well. Ciprofloxacin (5 µg/mL) was used as the positive and 1% DMSO as the negative control. The plates were incubated at 30 °C for 18–24 h. After the addition of 40 μL of 0.2 mg/mL of *ρ*-iodonitrotetrazolium violet (INT; Merck, Johannesburg, South Africa) dissolved in sterile distilled water, to each well, they were left to stand at room temperature for 4 h. The MIC was recorded as the lowest concentration of the extract that prevented the appearance of visible purple pigment growth of the organism after 24 h of incubation [42].

### 2.5. Preliminary Screening of Anti-Quorum Sensing (AQS) Activity

*Chromobacterium violaceum* was grown on LB agar. Thereafter, 5 mL of molten soft LB agar (0.3% *w*/*v*) was inoculated with 50 μL of *C. violaceum*, grown overnight in LB broth. The agar-culture mixture was immediately poured over the surface of pre-warmed LB agar plates as described [43]. After setting, wells were bored into the agar as before. For each extract, different concentrations (0.35, 1.75, 3.85 and 7.00 mg/mL) of the extract solutions were pipetted into the agar wells. The plates were incubated overnight at 30 °C and subsequently examined for violacein pigment production. Any AQS activity was evident by the formation of a colourless, opaque, but visible halo around the well, due to a loss of pigmentation. Eugenol (0.066 mg/mL) was used as the positive control, since it is a well-reported QS inhibitor. Dimethyl sulfoxide (1% DMSO) was used as the negative control.

### 2.6. Quantitative AQS Activity

Screening for AQS activity of the crude extracts was based on their ability to inhibit the production of the purple pigment violacein [44]. The strain was cultured aerobically in LB broth at 30 °C, with or without the addition of increasing concentrations (0.350–7.00 mg/mL) of plant extract. A 1.00 mL portion of the overnight culture of the biosensor strain was centrifuged (11,337× *g*, 10 min) to precipitate the insoluble violacein. The culture supernatant was discarded and the pellet was evenly re-suspended in 1.00 mL of DMSO (100%). The solution was again centrifuged (11,337× *g*, 10 min), to remove the cells, and the violacein was quantified by determining the optical density at 585 nm using a UV-Vis spectrophotometer. The percentage of violacein inhibition was calculated using the formula:

$$\mathrm{Percentage}\;\mathrm{violacein}\;\mathrm{inhibition}=\frac{\left({\mathrm{Control}}_{585\;\mathrm{nm}}{-\mathrm{Test}}_{585\;\mathrm{nm}}\right)}{Control_{585\;\mathrm{nm}}}\times 100$$

where Control represents the test bacteria without the presence of extract, and Test represents the bacteria exposed to the extract at various concentrations.

### 2.7. Identification of Major Active Compounds and Their AQS Activity

#### 2.7.1. High Performance Thin Layer Chromatography (HPTLC) Analysis

Preliminary identification of the active components present in thirteen crude extracts with AQS activity, namely *Apium graveolens* (ethyl acetate and methanol), *Armoracia rusticana* (aqueous), *Caspicum annum* (dichloromethane), *Glycyrrhiza glabra* (aqueous and methanol), *Melissa officinalis* (aqueous), *Rosmarinus officinales* (ethyl acetate), *Syzygium anisatum* (ethyl acetate and dichloromethane); *Syzygium aromaticum* (methanol) and *Thymus vulgaris* (ethyl acetate and methanol) was achieved by using a CAMAG (Switzerland) semi-automated HPTLC system and the indirect bio-autography assay (as described in Section 2.7.2) [16,17], with some modifications. The active extracts (3 µL) were spotted onto (20 × 10 cm) silica gel TLC plates (DC-Fertigfolien ALUGRAM1Xtra SIL G/UV_254_, Darmstadt, Germany) as 8 mm bands, 5 mm from the lower edge, using the automated TLC Sampler 4, fitted with a 25 µL Hamilton microsyringe and connected to a nitrogen supply. Thereafter, the plates were developed using toluene:ethyl acetate:methanol:formic acid (7:6:1.5:1) in an ADC2 development chamber, consisting of a glass twin-trough chamber (20 cm × 10 cm) with a metal lid. The developed plates were dried using the CAMAG TLC plate heater III. Two identical TLC plates were prepared: Plate 1 (not shown), the reference chromatogram was used to identify the presence of compounds, and determine their R*f* values, when visualised under white light and UV irradiation (254 and 366 nm), and Plate 2 used for bio-autography.

#### 2.7.2. HPTLC-Bio-Autography Assay

Developed HPTLC plates (Plate 2), to which the 13 AQS-active extract had been applied, were placed face-up in a sterile Petri dishes and an inoculum of *C. violaceum*, standardized to 0.5 McFarland standard equivalent in LB soft agar, was distributed evenly over the plates. After solidification of the medium, the Petri dishes were incubated at 30 °C for 24 h. A purple-coloured background, reflecting violacein production and QS by the bacterium, was observed on the plate. In contrast, the presence of pale, turbid zones indicated the presence of compounds in the extracts responsible for AQS activity. The R*f* values of the inhibition zones on the plate were compared with those determined from the reference chromatograms (Plate 1).

#### 2.7.3. Identification of the Target Compounds Using UHPLC-MS Analysis

After assaying the crude extracts, the ethyl acetate extract of *Apium graveolens* was singled out for identification of the major active compounds responsible for AQS activity, since the HPTLC-bio-autography plate displayed a prominent active spot. The target compound was identified using ultra performance liquid chromatography-mass spectrometry (UHPLC-MS), and then isolated using preparative-high performance liquid chromatography-mass spectrometry (prep-HPLC-MS). The activity of the pure compound was confirmed using the HPTLC-bio-autography assay.

The UHPLC-QToF-MS analysis was conducted using a Waters Acquity Ultra Performance Liquid Chromatography system equipped with a photodiode array (PDA) detector (Waters, Milford, MA, USA). The injection volume was 3 µL. Separation of the compounds in the extract was achieved on an Acquity UHPLC BEH C_18_ column (150 mm × 2.1 mm, i.d., 1.7 µm particle size, Waters) maintained at 40 °C. The mobile phase consisted of 0.1% formic acid in water (Solvent A) and 100% acetonitrile (Romil Ltd, Cambridge, UK) (Solvent B) at a flow rate of 0.4 mL/min. Gradient elution was applied as follows: 5% B to 90% B within 15 min, changed to 100% B over 3.5 min, maintaining for 1 min and then returning to the initial ratio within 0.5 min. Data were collected using MassLynx 4.1TM (Waters, Milford Massachusetts, MA, USA) chromatographic software. The UHPLC system was also interfaced with a Xevo G2QToF (Time-of-Flight) mass spectrometer. Both positive and negative ionisation modes were investigated, but the positive mode was selected since it yielded information-rich chromatograms. The desolvation temperature was set to 350 °C at a nitrogen flow rate of 500 L/h, and the source temperature was 100 °C. The capillary and cone voltages were set to 2900 and 45 V, respectively. Data were collected over the range *m/z* 100 to 1000.

#### 2.7.4. Isolation and Identification of the Active Compound

A Waters Autopurification system, equipped with a Waters photodiode array (PDA) detector (Model 2998) and a QDa mass spectrometer (Waters, Milford, MA, USA), was used for targeted fractionation of the ethyl acetate extract *of A. graveolens.* The injection volume was 500 µL. Separation was achieved on an XBridge Prep C18 column (250 x 19 mm i.d., 5 µm particle size, Waters) maintained at 40 °C. The mobile phase consisted of 0.1% formic acid in water (Solvent A) and acetonitrile (Solvent B) at a flow rate of 20 mL/min, Gradient elution was applied as follows: 5% B, kept constant for 1 min, increased to 40% B within 2 min, changed to 90% B over 12 min, and then to 95% B within 0.5 min, maintaining for 0.5 min, before returning to the initial ratio within 0.5 min. The following MS conditions were used: positive ionisation mode, probe temperature 600 °C, source temperature 120 °C, capillary voltage 800 V, cone voltages 10 V. Data were collected by MassLynx 4.1^TM^ (Waters, Milford, MA, USA) chromatographic software over the range *m/z* 100 to 600. Once isolated, the purity of the compound was determined using UHPLC-MS and UHPLC-photodiode array detection (PDA) and the activity was established using HPTLC-bio-autography against *C. violaceum* biomonitor strain. 

The isolated compound, dissolved in hexane, was identified using an Agilent 7683B gas chromatograph (GC), coupled to a mass spectrometer (MS, Agilent 5973). The sample, dissolved in hexane, was analysed on a 60 m polyethylene glycol capillary column (HP-Innowax, 250 μm diameter; 0.25 μm film thickness). Electron impact ionization was applied, with the ionisation voltage set at 70 eV, and mass spectra were collected over the range *m/z* 35–550. Three mass spectral libraries NIST, Flavor and Mass Finder 4 were used for comparison of the spectra.

## 3. Results and Discussion

### 3.1. Crude Extract Yields 

The extractants methanol and water resulted in higher extraction yields than DCM and ethyl acetate (Table 2). Methanol extracted the highest mass from *Mentha piperita* (19.5%) and *Glycyrrhiza glabra* (18.2%). This was followed by water for *Allium sativum*, *Capsicum annuum* and *Syzygium aromaticum* with 19.1%, 13.9% and 13.2% extract yield, respectively. Both DCM and ethyl acetate extracted the least from *Glycyrrhiza glabra* (1.3%), and *Armoracia rusticana Allium sativum*, *Cinnamomum zeylanicum* (1.7%). 

### 3.2. Qualitative Evaluation of AQS Activity 

The *C. violaceum* biomonitor strain was used for preliminary qualitative screening of extracts for AQS activity. Halos were observed on lawns of the *C. violaceum* as a result of either (i) inhibition of cell growth, seen as a clear halo, or (ii) quenching of QS signals, observed as a turbid halo. 

Varying degrees of bactericidal activity were observed (Table 3). The organism was susceptible to 12 of the 56 extracts tested, where the diameter of the clear inhibition zones were interpreted as follows: Susceptible (S) ≥ 15 mm, Intermediate (I) = 11–14 mm, and Resistant (R) ≤ 10 mm, as described by the Clinical and Laboratory Standards Institute [40]. Two of the aqueous, six of the methanol, two of the dichloromethane and two of the ethyl acetate extracts were highly active (zones ≥ 15 mm), indicating that the active constituents represented a wide polarity range. The methanol extract of *Glycyrrhiza glabra* (liquorice) was highly active towards *C. violaceum*, yielding 15 mm inhibition zones at all concentrations tested, suggesting that further dilutions would still be active against the organism. A concentration-dependent inhibitory effect was observed for the dichloromethane extract of *Cinnamomum zeylanicum* (cinnamon), the methanol, dichloromethane extract and ethyl acetate extracts of *Syzygium aromaticum* (clove), the ethyl acetate extract of *Armoracia rusticana* (horseradish)*,* and the aqueous extract of *Melissa officinalis* (lemon balm). *Chromobacterium violaceum* could be classified as susceptible to all these extracts, according to the zone diameters obtained, after exposure to the highest concentration (7.00 mg/mL). Furthermore, the organism displayed intermediate susceptibility towards most of the extracts over the range 7.00 to 0.350 mg/mL, with the exception of *Capsicum annuum* (cayenne pepper; aqueous extract), *Apium graveolens* (celery, methanol and ethyl acetate extracts), *Allium sativum* (garlic, methanol extract), *Melissa officinalis* (lemon balm, dichloromethane extract) and *Thymus vulgaris* (thyme, ethyl acetate extract), for which complete resistance was observed. The antimicrobial data corroborate results obtained in previous studies. For example, an aqueous extract of *Syzygium anisatum* (aniseed) produced significant antibacterial activity against common pathogenic Gram-negative and Gram-positive bacteria [45]. Antimicrobial assays with plant extracts (acetone, DMSO and buffered methanol) obtained from *Ruta graveolens* (common rue) and *Zingiber officinale* (ginger) exhibited inhibitory activities against *Bacillus cereus* strains [46].

### 3.3. Determination of MICs

The MICs determined for aqueous, methanol, dichloromethane and ethyl acetate extracts of the various herbs and spices against C. *violaceum* ranged from 2.00 to 4.00 mg/mL (Table 4). None of the results indicated noteworthy activity, which is regarded as MIC values < 1 mg/mL [47]. *Syzygium anisatum* (aniseed), *Syzygium aromaticum* (clove), *Glycyrrhiza glabra* (liquorice), *Melissa officinalis* (lemon balm) and *Thymus vulgaris* (thyme) extracts inhibited the growth of *C. violaceum* with MICs of 2.00 mg/mL for all four extracts, but this activity is not regarded as noteworthy (Table 4). 

When the antipathogenic potential of the plant extracts were evaluated, fading of the violet-colour of the violacein pigment in the area surrounding the wells indicated their possible AQS effect. Qualitative screening of AQS activity indicated that 13 of the tested plant extracts displayed the ability to interfere with, or inhibit violacein pigment production, as observed by opaque halo formation present immediately against the clear zone of dead bacteria (Figure S1A). Bacteria in these opaque zones were viable, but unable to secrete violacein, which is a reflection of a loss of QS ability. The aqueous extract of *Glycyrrhiza glabra* (liquorice) yielded the largest AQS inhibition zone (19 mm) (Table 4). Although the corresponding methanol extract exhibited a moderate MIC (2.00 mg/mL), it yielded a smaller AQS zone of inhibition (12 mm diameter) than the aqueous extract. The ethyl acetate (14 mm) and methanol (12–15 mm) extracts of *Apium graveolens* (celery) displayed similar AQS activity, but these extracts yielded MICs of 4.00 mg/mL (Table 4), reflecting poor bactericidal activity. This finding suggests that celery may be a good candidate for the isolation of active compounds with strong AQS, rather than strong bacteriocidal activity. *Melissa officinalis* (lemon balm, methanol extract) (9 mm) was the least active of those displaying AQS activity (Table 4). Most reported AQS agents demonstrate bactericidal effects, which often lead to AQS compounds being classified as antibacterial. However, in some cases compounds of natural origin yield poor MICs, yet have the ability to interfere with the QS-signalling mechanism at sub-MIC concentrations [21]. Packiavathy et al. [48] reported on the AQS ability of *Capparis spinosa* methanolic extract at 2 mg/mL, while Chenia [49] reported that the hexane, ethyl acetate and dichloromethane extracts of *Kigellia africana* displayed good AQS activity at 1.31 mg/mL, 1.97 mg/mL and 3.93 mg/mL, respectively.

### 3.4. Quantitative Evaluation of AQS Activity

**Figure 1:**
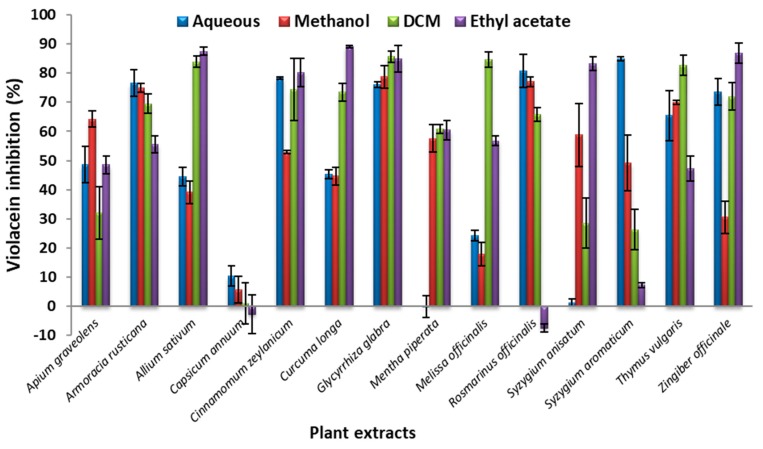
Percentage inhibition of violacein production after exposure of *Chromobacterium violaceum* to solvent extracts (0.7 mg/mL) of herbs and spice. Data presented as mean ± standard deviation (SD). Further percentage violacein inhibition results for plant extracts over a range of concentrations are provided as Supplementary data (Table S1).

To further validate the results obtained through the qualitative assay, all the extracts were evaluated in a quantitative AQS activity assay by extraction of violacein, following exposure of *C. violaceum* to the test substances. This was also done to confirm that the inhibition of violacein production was not due to a bactericidal effect. Varying levels of inhibition were observed with the four solvent extracts, most frequently in a dose-dependent manner, with most inhibitory effects (≥90%) recorded at the highest concentration (7.00 mg/mL). A sample displaying varying levels of inhibition after exposure to different extracts at 0.700 mg/mL is illustrated in Figure S1B. Eleven of the extracts were able to inhibit violacein production by more than 90% when applied at a concentration of 7.00 mg/mL, with the best performers identified as *Glycyrrhiza glabra* (liquorice, methanol extract, 97.3%), *Armoracia rusticana* (horseradish, aqueous extract, 97.2%), and *Rosmarinus officinalis* (rosemary, dichloromethane extract, 96.7%) (Table S1; Figure 1).

All four extracts of *Glycyrrhiza glabra* and *Melissa officinalis* (lemon balm), and three out of four extracts of *Mentha piperita* (mint), *Syzgium anisatum* (aniseed) and *Zingiber officinale* (ginger) inhibited the QS of *C. violacein* by more than 80%. Higher AQS activity was obtained for *Glycyrrhiza glabra*, *Armoracia rusticana*, *Rosmarinus officinalis*, *Thymus vulgaris*, *Curcuma longa* (turmeric), and *Melissa officinalis* in the quantitative violacein inhibition assay as compared to the qualitative assay. Only *Glycyrrhiza glabra* (aqueous and methanol extracts), *Armoracia rusticana* (aqueous extract), *Rosmarinus officinalis* (ethyl acetate extracts), *Thymus vulgaris* (methanol and ethyl acetate extracts), and *Melissa officinalis* (methanol extract) displayed potential AQS activity in the qualitative assay. However, almost all of the extracts exhibited some degree of AQS activity. *Curcuma longa* displayed no AQS activity when it was determined using the qualitative assay; however, more than 80% activity was observed for the ethyl acetate extract in the quantitative assay. This suggests that the qualitative agar well diffusion assay may not be an appropriate technique to judge AQS activity. A quantitative assay should be accepted as the standard to eliminate the discrepancy between the two methods.

It is noteworthy that, in many cases, dilution of the extracts to the lowest concentration (0.35 mg/mL) did not reduce the AQS activity in a dose-dependent manner. Dilution of *Glycyrrhiza glabra* and *Melissa officinalis* still yielded more than 74% inhibition of QS for all four extracts. The quorum sensing inhibitory effects of the aqueous extract of *Cinnamonum zeylanicum* and the ethyl acetate extract of *Curcuma longa* remained similar over the concentration range 0.350–7.00 mg/mL. Since the positive solvent controls and negative controls did not reveal any method inconsistencies, the reason for this result remains unclear. The best values were achieved for the aqueous (87.1 ± 7.3%) and dichloromethane (85.2 ± 3.5%) extracts of the two plants at 0.350 mg/mL, respectively. A similar pattern was established for the aqueous extract of *Rosmarinus officinalis* (82.2 ± 2.4%). The ethyl acetate extract (85.8 ± 2.4%) of *Circuma longa* also retained good activity when tested at the lowest concentration. 

Based on a literature search, no mention is made of the AQS potential of *Glycyrrhiza glabra* and *Melissa officinalis*. *Glycyrrhiza* species are ancient herbal medicines that are used worldwide and are known for their antimicrobial properties, due to the presence of triterpenoid saponins [50]. *Armoracia rusticana* produces allyl isothiocyanate, which was reported to be bactericidal towards antibiotic-resistant bacteria [51]. However, the reported AQS activity was attributed to another compound, identified as iberin, which completely inhibited the expression of *lasB*, without affecting bacterial growth in the *Pseudomonas aeruginosa* screen [52]. 

The results obtained in the current study indicate high AQS activity at sub-lethal concentrations as compared to other screenings based on ethnobotany. Several reports have documented the AQS activity of plant extracts against *Chromobacterium violaceum.* As an example, *Vanilla planifolia* (vanilla) has been reported to effectively inhibit the QS of *C. violaceum* [53]. The AQS activity of several fruit and spice extracts was attributed to interference with the AHL activity [54]. Raspberry, blueberry and grape extracts inhibited QS by 60%, 42% and 20%, respectively. Other herbs and spices were found to be more effective in reducing the AHL activity-mediated inhibition of violacein production (CV026), with basil (78%), thyme (60%) and *Brassica oleracea* (60%) being the most active, followed by rosemary, ginger and turmeric (40%) [54]. The methanolic extract of *Syzygium aromaticum* (clove) reportedly caused a strong inhibition of *C. violaceum* CV026 responding to AHL, thus inhibiting its violacein production [55]. Fifty medicinal plants were screened for their anti-pathogenic properties, of which only six of the plant extracts (*Conocarpus erectus*, *Chamaecyce, hypericifolia*, *Callistemon viminalis*, *Bucida burceras*, *Tetrazygia bicolor* and *Quercus virginiana*) demonstrated good AQS activities [56]. It was also reported [39] that *Apium graveolens*, *Thymus vulgaris* and *Rosmarinus officinalis* essential oils had no AQS activity. However, essential oils of *Syzygium aromaticum*, *Cinnamomum verum* and *Mentha piperita* displayed good AQS activity against CV12472 and CV026 in the presence of C_6_-AHL [39].

### 3.5. Identification and Isolation of Secondary Metabolites in Celery

Amongst the plant extracts tested, *Apium graviolens* ethyl acetate extract produced only turbid halos attributed to QS signal molecule quenching, with no effect on bacterial cell growth. This observation, together with paucity in the published literature on the AQS properties of celery, prompted a more in-depth investigation of the chemistry of *Apium graveolens* using bio-autography as a guide to identify compounds with AQS activity. 

**Figure 2:**
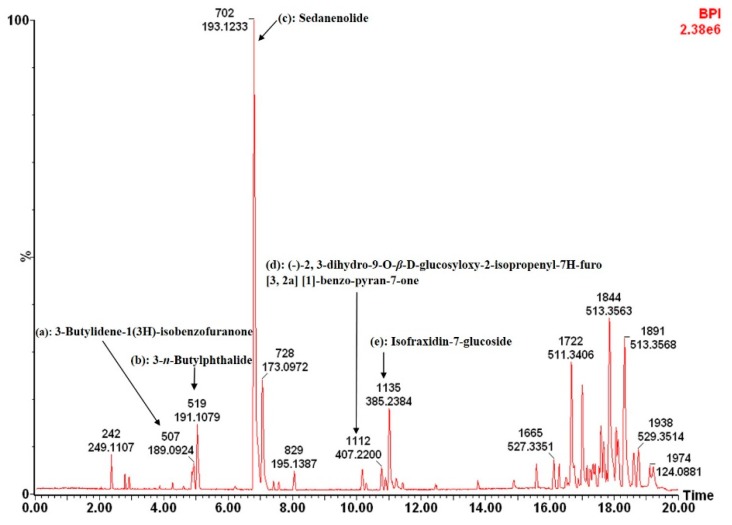
Ultra performance liquid chromatography-mass spectrometry (UHPLC-MS) chromatogram of the ethyl acetate crude extract of *Apium graveolens* indicating the presence of compounds identified using mass spectral data corresponding to positive mode.

The UHPLC-MS profile of the ethyl acetate crude extract revealed seven compounds, which were identified as (a) 3-butylidene-1(3H)-isobenzofuranone [56]; (b) 3-*n*-butylidene-phthalide [57]; (c) sedanenolide [57]; (d) (-)-2,3-dihydro-9-*O*-*β*-D-glucosyloxy-2-isopropenyl-7H-furo[3,2a][1]- benzo-pyran-7-one [58]; (e) isofraxidin-7-glucoside [59] and sedanenolide (3-*n*-butyl-4,5-dihydrophthalide), after comparison of their spectral data with data reported in literature (Figure 2). Phthalide compounds have a broad distribution and are prevalent in *Apium graveolens*. As example, Tang et al. [57] documented two major phthalide compounds, namely 3-*n*-butylphthalide (sedanolide) and 3-*n*-butyl-4,5-dihydrophthalide (sedanenolide) in *Apium graveolens* oil. These butylphthalides have been recognised for their physiological activities including anti-inflammatory, anti-carcinogenic and insecticidal effects [60].

**Figure 3:**
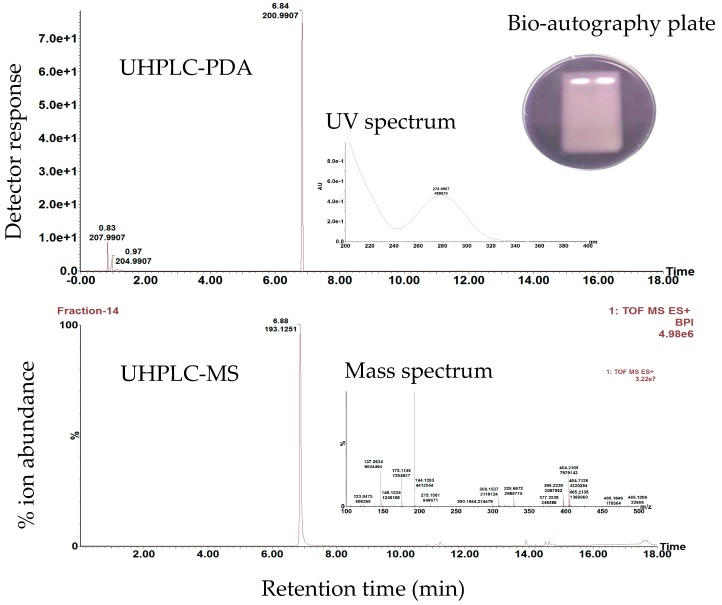
Ultra performance liquid chromatography (UHPLC)-photodiode array (PDA) and UHPLC-mass spectrometry (MS) chromatograms of sedanenolide, isolated from the ethyl acetate crude extract of *Apium graveolens.* The ultraviolet absorbance spectrum and mass spectrum are also provided. The high performance thin layer chromatography (HPTLC)-bio-autography plate of pure sedanenolide (in duplicate) against *Chromobacterium violaceum* is also indicated with the white zones confirming the anti-quorum sensing activity of the compound.

In the present study, only sedanenolide was successfully isolated from *Apium graveolens* extract using prep-HPLC-MS, which enables the targeted isolation of a selected ion. The purity (97%) of the compound was determined using UHPLC-MS and UHPLC-PDA (Figure 3), while the identity was confirmed as sedanenolide using GC-MS. When the compound was subjected to a HPTLC-bio-autography assay, AQS potential was observed as indicated by the zones evident in Figure 3. The AQS activity of sedanenolide probably involves the inhibition of the QS-related factor of *C. violaceum*, due to its structural similarity to the AHL class of QS-signal molecules. The mechanism of inhibition includes the compound competing and interfering with the activity of the signal molecules, due to their structural similarity. By binding to *cvi*R, which encodes the enzyme for N-hexanoyl-L-homoserine lactone (C6-HSL) homologues, a rapid turnover of these proteins or alteration to their synthesis occurs, by promoting the degradation of CviR receptor proteins that bind the signal molecules [61]. By so doing, deleted or inactivated *cvi*I interferes with, and reduces, QS-controlled violacein production by *C. violaceum* (12472). Several natural compounds have been demonstrated to behave as antagonists. These include halogenated acyl-furanones, which are structurally similar to AHLs, and were derived from the marine alga *Delisea pulchra* [62]. These compounds displace the 3-oxo-C6-HSL signal from its cognate LuxR receptor protein, thereby inhibiting the QS-mediated gene expression.

## 4. Conclusions

In this study, a better understanding of the health impact of herbs and spices was gained by investigating their AQS potential. Of the 56 plant extracts screened, several displayed both antimicrobial and AQS activities. All 14 herbs and spices assessed have potential to serve as either antimicrobial or antipathogenic agents. The quantitative AQS assay should be considered as a standard technique for screening botanicals for AQS agents. Based on the quantitative violacein inhibition, almost all extracts of different polarities displayed potential AQS activity, with the exception of *Syzgium anisatum* (ethyl acetate) and *Zingiber officinale* (methanol) extract, which caused less than 40% inhibition. The ethyl acetate extract of *Apium graveolens* was selected as the subject for further study since it demonstrated poor bactericidal activity towards *C. violaceum*, but inhibited the production of violacein by the bacterium. The extract and active compounds of celery have the potential to serve in the development of antipathogenic drugs and contribute to combating bacterial diseases, without adding to the burden of antibiotic resistance. Celery will have a greater advantage than any other botanicals for human use since it has been used safely for a long time and is edible. This study substantiates the potential of dietary phytochemicals as a source of anti-pathogenic compounds and highlights the importance of investigating understudied plants, rather than the traditional medicinal plants. Culinary herbs and spices present exciting opportunities for health promotion by contributing anti-oxidant, antimicrobial, pharmaceutical and nutritional properties. This study has contributed valuable information on the AQS properties of common culinary herbs and spices, to further the development of next-generation antipathogenic or antivirulence agents involved in inactivating signalling molecules that control pathogenicity.

## Figures and Tables

**Table 1:** Herbs and spices selected for screening for anti-quorum sensing (AQS) potential.

Species Name	Family Name	Common Name	Medicinal Use	Reference
*Allium sativum*	Amaryllidaceae	Garlic	Treatment of various disorders such as respiratory ailments, asthma, pneumonia, diabetes, cardiovascular disorders and rheumatism	[22,23]
*Apium graveolens*	Apiaceae	Celery	Treat toothache, diarrhea, hypertension and-pulmonary disease. Used as stimulant, cardiac tonic, carminative, diuretic and antiseptic	[24,25]
*Armoracia rusticana*	Brassicaceae	Horse-radish	Cough, lung and heart disease, diuretic, digestion, wounds, shortness of breath, stomach problems, bronchitis, headache, high blood pressure and rheumatism	[26]
*Capsicum annuum*	Solanaceae	Cayenne pepper	Use in traditional medicine to alleviate gastric ulcers, rheumatism, alopecia, toothache and diabetes	[27,28]
*Cinnamomum zeylanicum*	Lauraceae	Cinnamon	Benefit for common cold, cardiovascular, neurodegenerative diseases and gastrointestinal disorders	[4]
*Curcuma longa*	Zingiberaceae	Turmeric	Anti-inflammatory and for the treatment of jaundice, menstrual difficulties, hematuria, hemorrhage, and colic. Applied topically for urticaria and skin allergy, viral hepatitis, inflammatory conditions of joints, sore throat and wounds	[29]
*Glycyrrhiza glabra*	Fabaceae	Liquorice	Used in medicines for its unique and diverse pharmacological properties *viz*., antiviral, anticancer, anti-ulcer, anti-diabetic, anti-inflammatory, anti-oxidant, immuno-stimulant, anti-allergenic	[30]
*Melissa officinalis*	Lamiaceae	Lemon balm	Treat infections of Herpes simplex	[31]
*Mentha piperita*	Lamiaceae	Peppermint (wild)	Used to treat coughs, bronchitis, inflammation of oral mucosa and throat, pulmonary tuberculosis, digestive complaints such as colic in infants, flatulence, diarrhea, indigestion, nausea, morning sickness and anorexia	[32]
*Rosmarinus officinalis*	Lamiaceae	Rosemary	Relief pain in renal colic and dysmenorrhoea, and as antispasmodic diuretic, antipyretic and as a mood stabilizer	[33]
*Syzygium anisatum*	Myrtaceae	Aniseed	Sedative and stimulant in cough medicines	[34]
*Syzygium aromaticum*	Myrtaceae	Cloves	Used to treat indigestion, flatulence, nausea, vomiting, diarrhea, cough, infertility, warts, hernias, ringworm, wounds, toothaches, athletes foot and other fungal infections	[34,35,36]
*Thymus vulgaris*	Lamiaceae	Thyme	Bronchial asthma, inflammatory affection, hepatotoxicity, atherosclerosis, ischaemic heart disease, cataracts, cancer, insufficient sperm mobility	[37]
*Zingiber officinale*	Zingiberaceae	Ginger	Used for cold-induced diseases, nausea, asthma, cough, colic, heart palpitation, swelling, dyspepsia, less appetite and rheumatism	[38]

**Table 2:** Crude extract yields (% *w/w*) obtained after extraction of herbs and spices with different solvents.

Plants	Solvents			
	Aqueous	Methanol	DCM	Ethyl Acetate
*Allium sativum*	19.1%	10.5%	1.7%	1.7%
*Apium graveolens*	3.0%	4.1%	10.0%	7.5%
*Armoracia rusticana*	8.3%	8.6%	6.0%	1.3%
*Capsicum annuum*	13.9%	6.4%	6.7%	3.3%
*Cinnamomum zeylanicum*	4.0%	3.3%	3.1%	1.7%
*Curcuma longa*	3.3%	7.5%	2.8%	2.2%
*Glycyrrhiza glabra*	5.6%	18.2%	4.8%	1.3%
*Melissa officinalis*	8.3%	11.1%	6.6%	10.8%
*Mentha piperita*	10.8%	19.5%	1.7%	4.3%
*Rosmarinus officinalis*	8.2%	10.6%	8.4%	6.0%
*Syzygium anisatum*	10.1%	4.8%	5.4%	5.3%
*Syzygium aromaticum*	13.2%	6.4%	5.6%	3.8%
*Thymus vulgaris*	11.4%	11.5%	6.4%	6.1%
*Zingiber officinale*	7.0%	6.4%	3.3%	5.8%

**Table 3:** Antibacterial activity of a range of concentrations of herbs and spices as determined using the agar well diffusion method.

Plant Pecies	Zone Diameters (mm) and Associated Susceptibility Phenotypes															
	Aqueous Extract				Methanol Extract				Dichloromethane Extract				Ethyl Acetate Extract			
	Concentration (mg/mL)															
	0.350	1.75	3.85	7.00	0.350	1.75	3.85	7.00	0.350	1.75	3.85	7.00	0.350	1.75	3.85	7.00
*Allium sativum*	11(I)	11(I)	12(I)	13(I)	10(R)	10(R)	10(R)	10(R)	11(I)	12(I)	12(I)	12(I)	11(I)	11(I)	12(I)	12(I)
*Apium graveolens*	10(R)	10(R)	11(I)	11(I)	0(R)	0(R)	0(R)	0(R)	11(I)	12(I)	12(I)	12(I)	0(R)	0(R)	0(R)	0(R)
*Armoracia rusticana*	0(R)	10(R)	11(I)	11(I)	12(I)	11(I)	11(I)	11(I)	12(I)	13(I)	13(I)	12 (I)	10(R)	11(I)	12(I)	14(S)
*Capsicum annuum*	10(R)	10(R)	10(R)	10(R)	12(I)	12(I)	13(I)	13(I)	11(I)	12(I)	12(I)	13(I)	11(I)	12(I)	12(I)	13(I)
*Cinnamomum zeylanicum*	11(I)	12(I)	11(I)	11(I)	11(I)	11(I)	12(I)	12(I)	11(I)	12(I)	12(I)	14(S)	11(I)	12(I)	13(I)	12(I)
*Curcuma longa*	10(R)	11(I)	12(I)	12(I)	10(R)	11(I)	12(I)	12(I)	10(R)	11(I)	12(I)	12(I)	10(R)	11(I)	12(I)	12(I)
*Glycyrrhiza glabra*	10(R)	11(I)	12(I)	13(I)	15(S)	15(S)	15(S)	15(S)	10(R)	10(R)	10(R)	11(I)	13(I)	13(I)	13(I)	13(I)
*Melissa officinalis*	11(I)	12(I)	15(S)	15(S)	11(I)	11(I)	11(I)	11(I)	10(R)	10(R)	10(R)	10(R)	11(I)	11(I)	11(I)	11(I)
*Mentha piperita*	11(I)	12(I)	12(I)	12(I)	10(R)	11(I)	12(I)	12(I)	11(I)	12(I)	12(I)	12(I)	11(I)	12(I)	12(I)	12(I)
*Rosmarinus officinalis*	10(R)	11(I)	11(I)	12(I)	11(I)	11(I)	13(I)	13(I)	11(I)	11(I)	13(I)	13(I)	11(I)	11(I)	10(R)	10(R)
*Syzygium anisatum*	12(I)	13(I)	13(I)	12(I)	11(I)	12(I)	13(I)	16(S)	11(I)	11(I)	13(I)	14(S)	11(I)	11(I)	11(I)	14(S)
*Syzygium anisatum*	11(I)	12(I)	12(I)	12(I)	11(I)	12(I)	11(I)	11(I)	0(R)	11(I)	11(I)	11(I)	12(I)	12(I)	12(I)	12(I)
*Thymus vulgaris*	10(R)	10(R)	12(I)	13(I)	10(R)	10(R)	10(R)	12(I)	11(I)	11(I)	11(I)	11(I)	10(R)	10(R)	10(R)	10(R)
*Zingiber officinale*	11(I)	12(I)	13(I)	13(I)	12(I)	12(I)	12(I)	14(S)	12(I)	13(I)	13(I)	13(I)	12(I)	13(I)	13(I)	13(I)
CIP5 ^#^	31(S)				30(S)				30(S)				30(S)			
Eugenol	12(I)				22(S)				24(S)				28(S)			

CIP5 ^#^: ciprofloxacin (5 µg/mL). R, I and S denote Resistant, Intermediate Susceptibility and Susceptible.

**Table 4:** Minimum inhibitory concentrations (MICs) (mg/mL) determined using the broth microdilution method, and anti-QS activities using the agar well diffusion method of solvent extracts of various herbs and spices.

Plants	MIC (mg/mL) against CV12472				Anti-QS Activities	
	Extracts				Active Plant Extract	AQS Zone of Inhibition (mm)
	Aqueous	Methanol	DCM	Ethyl Acetate		
*Allium sativum*	4	4	4	4	NA	NA
*Apium graveolens*	4	4	4	4	ethyl acetate; methanol	14; 12–15
*Armoracia rusticana*	2	4	4	4	aqueous	12–14
*Capsicum annuum*	4	4	4	4	DCM	13–14
*Cinnamomum zeylanicum*	4	2	2	2	NA	NA
*Curcuma longa*	4	2	2	2	NA	NA
*Glycyrrhiza glabra*	2	2	2	2	aqueous; methanol	19; 12
*Melissa officinalis*	2	2	2	2	methanol	9
*Mentha piperita*	4	4	4	4	NA	NA
*Rosmarinus officinalis*	2	2	4	4	ethyl acetate	12–13
*Syzygium anisatum*	2	2	2	2	ethyl acetate; DCM	14; 13
*Syzygium aromaticum*	2	2	2	2	methanol	13
*Thymus vulgaris*	2	2	2	2	ethyl acetate; methanol	13; 12
*Zingiber officinale*	4	2	2	2	NA	NA

DCM = dichloromethane; NA = not active.

## Supplementary Materials

The following are available online at https://www.mdpi.com/2072-6643/11/4/739/s1, Table S1: Quantification of quorum sensing inhibitory effect of different plant extracts on violacein production at different concentrations (0.350–7.00 mg/mL), Figure S1: Representation of the agar well diffusion assay used to screen various plant extracts against *Chromobacterium violaceum* ATCC 12472.

## Abbreviations

The following abbreviations were used in this manuscript:

AHL	acylhomoserine lactone
AQS	anti-quorum sensing
ATCC	American Type Culture Collection
DCM	Dichloromethane
DMSO	dimethyl sulfoxide
GRAS	Generally Regarded As Safe
HPTLC	high performance thin layer chromatography
I	Intermediate
INT	*p*-iodonitrotetrazolium violet
LB	Luria Bertani
LC-MS	liquid chromatography-mass spectrometry
MIC	minimum inhibitory concentration
NA	not active
PDA	photodiode array
prep-HPLC-MS	preparative high performance liquid chromatography-mass spectrometry
QS	quorum sensing
R	Resistant
R*f*	retardation factor
S	Susceptible
UHPLC-QToF-MS	ultra high performance liquid chromatography-quadrupole Time-of-Flight-mass spectrometry
USFDA	United States food and Drug Administration
