# Supplementary material for: Exploring Common Culinary Herbs and Spices as Potential Anti-Quorum Sensing Agents
Source: Nutrients. 2019 Mar 29;11(4):739. doi: 10.3390/nu11040739 (PMC6521156; doi:10.3390/nu11040739)
Supplement: Supplementary file 1 [file nutrients-11-00739-s001.pdf]

Table S1: Quantification of quorum sensing inhibitory effect of different plant extracts on violacein production at different concentrations (0.350–7.00 mg/mL). Results are expressed in percentage of violacein inhibition. Data presented as mean ± SD.

Extract	Concentration (mg/mL)							
	**0.350**	**0.700**	**1.75**	**2.80**	**3.85**	**4.90**	**5.95**	**7.00**
	Allium sativum							
**Aqueous**	47.8±1.9	44.4±3.1	40.6±1.8	72.7±1.5	72.2±1.3	72.8±0.8	68.1±2.4	78.2±2.8
**Methanol**	20.7±3.4	39.2±3.9	31.0±1.5	35.8±6.4	40.2±7.3	42.7±2.3	43.5±1.8	49.3±3.8
**Dichloromethane**	64.6±0.7	84.2±1.91	83.8±5.1	83.6±1.9	79.8±0.9	76.1±4.4	73.1±2.1	75.9±0.7
**Ethyl acetate**	34.2±4.4	68.1±1.4	87.5±3.3	85.5±1.8	91.6±3.0	93.3±2.3	88.5±7.5	90.2±4.3
	Apium graveolens							
**Aqueous**	28.5±5.1	48.6±6.2	50.7±6.0	65.7±5.3	55.3±1.2	56.4±3.6	56.4±3.6	55.7±1.4
**Methanol**	64.9±1.3	64.2±2.8	64.5±1.0	74.1±3.1	86.0±2.7	87.8±2.8	86.8±1.9	88.4±4.4
**Dichloromethane**	29.1±4.3	32.1±8.9	33.6±7.9	38.1±7.3	47.6±6.5	49.8±7.8	58.1±5.1	44.5±2.4
**Ethyl acetate**	20.1±2.3	48.5±3.0	68.7±4.2	70.6±6.8	75.1±5.8	80.5±4.4	75.6±5.6	74.6±4.6
	Armoracia rusticana							
**Aqueous**	69.3±8.9	76.5±4.5	78.5±3.3	73.9±5.8	88.4±3.5	96.8±1.4	95.6±1.1	97.2±1.6
**Methanol**	68.4±4.5	74.9±1.4	67.9±4.9	74.5±3.7	80.2±1.4	80.5±3.0	79.6±3.2	88.6±3.0
**Dichloromethane**	73.2±4.3	69.5±3.3	63.2±2.1	50.7±3.6	44.9±2.8	45.2±2.4	46.9±2.1	53.9±4.4
**Ethyl acetate**	54.9±2.2	55.6±2.9	55.1±2.2	69.3±2.7	56.4±3.0	50.2±0.4	55.6±4.1	51.9±4.3
	Capsicum annuum							
**Aqueous**	10.3±5.1	17.0±3.4	26.0±5.7	30.9±5.4	49.5±6.9	45.0±8.9	52.1±2.3	76.1±6.9
**Methanol**	5.57±6.38	14.4±4.6	17.9±4.4	30.7±6.3	33.6±4.4	34.7±2.3	41.8±1.0	42.7±4.1
**Dichloromethane**	1.03±6.61	3.15±7.09	8.42±4.47	18.7±5.0	32.3±6.8	40.3±7.4	46.2±3.8	48.7±2.7
**Ethyl acetate**	6.00±8.27	-2.78±6.57	-15.4±8.2	14.5±12.4	40.7±3.5	56.7±1.2	53.5±1.6	69.9±5.8
	Cinnamomun zeylanicum							
**Aqueous**	79.7±4.4	78.3±0.5	81.6±1.0	74.8±1.2	75.6±2.7	73.3±0.9	71.4±1.2	69.4±4.0
**Methanol**	55.2±2.6	52.9±0.7	20.9±1.8	32.2±6.7	43.2±0.9	44.3±3.0	43.5±2.4	41.6±9.4
**Dichloromethane**	67.3±5.8	74.4±10.7	42.5±4.7	70.3±5.1	72.8±5.4	69.7±1.6	72.5±5.0	59.8±4.3
**Ethyl acetate**	60.7±3.9	80.2±4.9	83.8±1.6	97.1±0.5	89.0±5.2	91.2±0.9	86.7±6.3	81.9±2.3
	Curcuma longa							
**Aqueous**	32.0±5.0	45.3±1.5	55.0±1.7	77.6±1.7	78.6±2.1	76.8±1.3	82.7±0.9	69.1±4.0
**Methanol**	60.2±8.2	44.6±3.1	67.2±2.2	83.9±2.2	91.3±2.0	86.3±1.2	84.1±2.5	81.0±3.7
**Dichloromethane**	71.9±1.5	73.4±3.1	77.0±4.1	76.4±0.8	86.0±0.3	89.8±1.7	91.6±3.2	87.1±1.9
**Ethyl acetate**	85.8±2.4	89.1±0.5	86.8±1.0	85.3±2.0	82.0±2.3	83.6±0.6	83.0±0.9	87.5±3.3
	Glycyrrhiza glabra							
**Aqueous**	87.1±7.3	76.1±0.9	84.3±0.7	85.2±4.5	84.8±3.2	84.8±3.1	75.5±1.8	91.2±4.0
**Methanol**	78.7±3.8	79.0±3.8	85.8±0.8	95.0±0.2	89.5±1.8	97.3±3.7	94.9±3.6	94.0±3.5
**Dichloromethane**	85.2±3.5	85.6±2.0	80.6±1.8	86.1±2.9	91.2±2.3	90.7±3.4	88.4±0.6	94.4±1.6
**Ethyl acetate**	74.1±5.5	84.8±4.5	87.0±3.9	87.75±1.36	89.69±1.36	82.7±0.6	84.7±2.2	85.6±2.4
	Mentha piperita							
**Aqueous**	-0.12±5.36	4.93±3.61	13.4±6.3	19.69±4.78	28.66±7.32	23.6±3.0	61.7±1.2	85.6±2.4
**Methanol**	47.0±5.1	57.6±4.8	64.2±2.1	79.52±2.32	81.64±4.16	83.3±4.4	87.9±1.2	95.0±4.0
**Dichloromethane**	53.6±4.3	60.8±1.6	46.0±1.5	64.48±4.44	73.03±3.27	75.3±6.1	68.8±1.0	68.7±3.9
**Ethyl acetate**	67.6±1.6	60.5±3.3	63.2±2.4	75.57±0.64	71.21±1.36	83.6±0.4	87.8±2.0	91.4±2.6
	Melissa officinalis							
**Aqueous**	76.1±7.3	87.2±0.9	84.3±0.7	85.2±4.5	84.8±3.2	84.8±3.1	75.5±1.8	91.2±4.0
**Methanol**	78.7±3.8	78.7±3.8	85.8±0.8	95.0±0.2	89.5±1.8	97.3±3.7	94.9±3.6	94.0±3.5
**Dichloromethane**	85.2±3.5	85.6±2.0	80.6±1.8	86.1±2.94	91.2±2.3	90.8±3.4	88.4±0.6	94.4±1.6
**Ethyl acetate**	74.1±5.5	84.8±4.5	87.0±3.9	87.8±1.4	89.7±1.4	82.7±0.6	84.7±2.2	85.6±2.4
	Rosmarinus officinalis							
**Aqueous**	82.2±2.4	80.7±5.6	61.5±0.9	78.1±1.8	89.3±5.2	88,8±3.6	84.4±4.4	81.8±3.7
**Methanol**	54.1±2.6	54.3±1.6	69.0±4.8	68.4±3.6	67.6±1.6	71.0±2.1	69.2±3.3	77.0±3.2
**Dichloromethane**	48.7±4.8	65.9±2.3	68.3±1.8	69.1±11.4	69.3±8.3	90.4±5.3	96.7±1.0	96.8±0.6
**Ethyl acetate**	-7.51±9.58	6.15±1.41	17.0±5.9	39.7±12.6	46.5±10.4	64.6±3.9	65.5±5.6	70.1±11.6
	Syzygium anisatum							
**Aqueous**	7.57±1.64	1.27±0.27	17.2±2.4	33.9±4.3	35.7±4.7	24.7±2.1	23.0±2.6	24.1±1.3
**Methanol**	53.5±1.2	58.8±10.9	55.9±5.53	67.1±9.0	68.8±4.3	71.6±4.3	81.7±1.2	87.5±3.5
**Dichloromethane**	31.3±3.8	28.5±8.5	38.1±3.1	51.0±6.1	57.5±4.7	58.5±0.8	65.5±1.5	87.0±2.8
**Ethyl acetate**	58.7±3.1	61.1±2.3	63.7±1.7	66.3±3.4	72.1±2.1	79.2±7.7	83.2±2.0	84.1±4.6

				Syzygium aromaticum				
**Aqueous**	64.3±3.3	85.0±0.7	86.2±1.2	84.7±2.1	88.6±2.7	92.1±0.4	88.4±1.0	88.7±2.3
**Methanol**	49.1±3.5	54.1±9.6	58.8±7.0	69.6±5.7	71.9±6.4	73.3±1.1	79.4±4.2	84.8±4.1
**Dichloromethane**	-11.6±3.9	26.2±6.9	35.3±4.5	35.5±1.5	44.4±5.1	47.5±3.9	57.6±2.2	65.3±3.2
**Ethyl acetate**	7.27±2.11	11.2±0.8	21.6±7.3	38.4±2.4	68.1±4.3	68.1±4.1	85.45±1.00	91.8±1.6
				Thymus vulgaris				
**Aqueous**	47.5±8.8	65.4±8.7	50.2±6.2	71.2±7.7	75.7±5.6	89.5±4.3	89.15±4.99	90.5±1.4
**Methanol**	51.8±6.7	50.5±0.8	69.9±1.1	86.9±1.9	91.7±3.58	92.4±4.1	88.64±2.44	94.5±4.8
**Dichloromethane**	60.7±3.2	71.8±3.4	82.7±0.9	81.7±4.2	80.61±2.65	86.7±1.3	82.67±0.82	87.4±3.4
**Ethyl acetate**	19.0±9.3	18.4±4.3	21.3±2.1	25.0±3.7	33.94±4.75	36.4±6.2	47.21±3.05	66.8±8.5
				Zingiber officinale				
**Aqueous**	67.3±2,3	73.5±4.6	73.8±3.8	79.4±3.1	78.6±3.2	78.8±3.1	80.7±2.9	85.7±2.0
**Methanol**	12.4±5.1	16.1±5.5	18.4±10.9	20.2±0.0	22.0±0.5	21.0±4.6	20.5-±10.7	27.3±1.3
**Dichloromethane**	60.2±4.1	64.8±4.7	65.9±1.6	72.0±1.9	74.5±1.1	76.7±3.9	80.5±0.8	82.1±2.4
**Ethyl acetate**	75.0±6.10	71.8±3.5	75.1±5.9	77.0±5.4	77.9±4.3	78.7±2.2	81.5±2.9	86.8±1.0

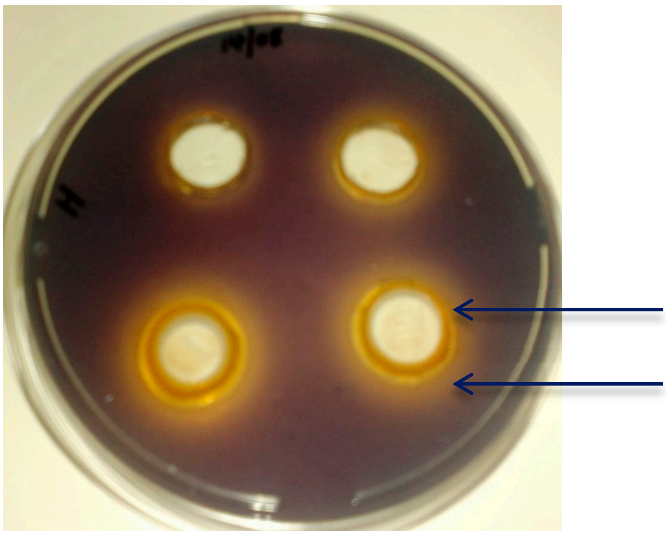

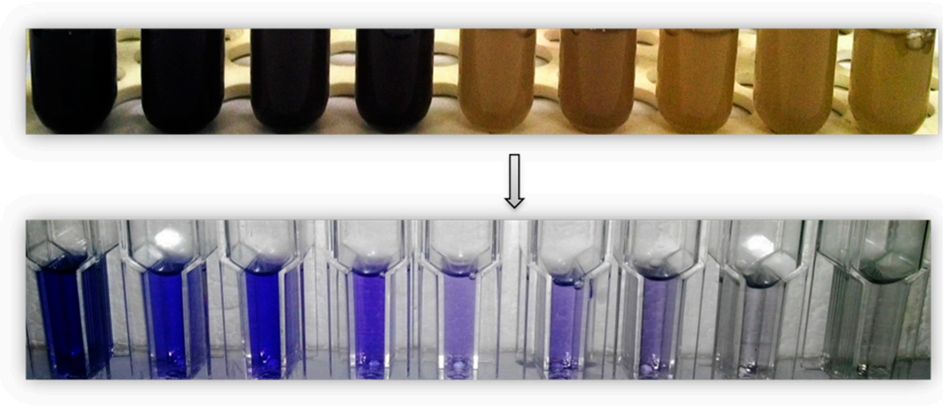

Fig. **S1:** Representation of the agar well diffusion assay used to screen various plant extracts against *Chromobacterium violaceum* ATCC 12472. The upper image indicates both the antibacterial activity (clear zones) and quorum sensing inhibition (halo zones). Quantification of inhibition of violacein production (lower image), following exposure of the bacterium to increasing concentrations of extracts. A concentration-dependent inhibitory effect of *Apium graveolens* ethyl acetate extract is evident.
